# The Impact of Preoperative Duration of Fasting on the Intravascular Volume Status of Children Older than 5 Years of Age: A Prospective, Observational Study

**DOI:** 10.4274/TJAR.2025.251934

**Published:** 2025-10-14

**Authors:** Beliz Bilgili, Tümay Umuroğlu

**Affiliations:** 1 Marmara University Faculty of Medicine Department of Anaesthesiology and Intensive Care, İstanbul, Türkiye

**Keywords:** Cardiac index, fasting time, fluid responsiveness, intravascular volume, paediatric anaesthesia, passive leg raising

## Abstract

**Objective:**

Preoperative fasting is a common practice aiming to reduce the risk of pulmonary aspiration during anaesthesia. It is advised to avoid fasting times longer than 6 hours in all children, whenever possible. Prolonged fasting can be uncomfortable for children and may lead to dehydration and other negative outcomes. The primary outcome of the study was the relationship between preoperative duration of fasting and cardiac index (CI) variability, used as a surrogate for intravascular volume status after the induction of anaesthesia, in paediatric patients undergoing surgery.

**Methods:**

Prospective, observational study that included patients over 5 years of age, scheduled for surgery. Passive leg-raising-induced CI variability was evaluated for fluid responsiveness and intravascular volume after anaesthesia induction. Patients were termed fluid responders (Rs) if an increase in CI of >10% was obtained after passive leg raising, and non-responders (NRs) if the CI variability was <10%. CI and aortic peak velocity (V_peak_) were measured through the suprasternal notch via an ultrasonic cardiac output monitor.

**Results:**

There were 32 Rs and 53 (NRs). The mean duration of fasting for Rs was 11.53±2.61, while NR had a mean duration of fasting of 10.6±2.93 hours, showing an insignificant difference. Aortic V_peak_ change was significantly higher in Rs (0.24±0.17) compared to NRs (0.03±0.13) (*P* < 0.001). Duration of fasting showed no significant correlation with CI variability and peak aortic velocity.

**Conclusion:**

With this study method, it was observed that preoperative fasting time had no effect on intraoperative intravascular volume.

**Main Points**

• Paediatric patients have higher insensible losses due to relatively increased surface area, increased respiratory rate, and greater metabolic rate, and are therefore more prone to become hypovolemic in the perioperative period.

• Prolonged fasting can be uncomfortable for children and may lead to dehydration and other negative outcomes.

• Even extensively prolonged preoperative fasting times had no significant impact on intravascular volume status in children over 5 years of age under anaesthesia.

## Introduction

Preoperative fasting is a common practice aiming to reduce the risk of pulmonary aspiration during anaesthesia. Guidelines typically recommend specific fasting times including clear fluids up to 2 hours, breast milk up to 4 hours, and a light meal up to 6 hours before elective procedures.^1^ It is advised to avoid prolonged fasting times longer than 6 hours in all children, whenever possible.^2^ Nevertheless, the actual duration of fasting is frequently much longer than advised, including clear fluids abstinence.^3, 4^ This undesirable situation is mostly inevitable and unpredictable, occurring because of delays in scheduling or overly cautious practices. Paediatric patients have higher insensible losses due to increased surface area, increased respiratory rate, and greater metabolic rate, and are therefore more prone to become hypovolemic during the perioperative setting.^5^ Prolonged fasting can be uncomfortable for children and may lead to dehydration and other negative outcomes.^6^ Adequate hydration can prevent dehydration-related complications and delayed recovery.^7^

As prolonged fasting may lead to dehydration that may predispose paediatric patients to hemodynamic instability during anaesthesia it must be carefully managed. There may be a relationship between the duration of preoperative fasting and the occurrence of hypotension, which is a potential indicator of intravascular volume depletion. Fluid fasting times longer than 6 hours were associated with an increased risk of postinduction hypotension, during surgical preparation, although this association seemed to be non-linear.^8^ However, another study found no association between fasting duration and hypotension.^9^ The literature presents conflicting findings on this subject. Additionally, it should be noted that blood pressure is generally maintained in children with hypovolemia, and the lack of age-specific definitions for hypotension limits the ability of blood pressure to accurately reflect intravascular volume status.

The impact of preoperative fasting time on intravascular volume in paediatric patients remains a subject of debate and investigation. Understanding the impact of fasting time on intravascular volume status is crucial for optimizing perioperative care in paediatric patients. Recent research has focused on dynamic parameters and functional hemodynamic monitoring techniques to assess intravascular volume and fluid responsiveness. Since conventional static measures (e.g., blood pressure, urine output) may not accurately reflect real-time intravascular volume shifts, dynamic parameters such as cardiac index (CI) variability and aortic peak velocity (V_peak_) serve as primary indicators of volume status. CI represents cardiac output (CO) normalized to body surface area, providing a better assessment of circulatory adequacy in paediatric patients than CO alone. Dynamic changes in CI following a preload challenge [such as passive leg raising (PLR)] offer insight into fluid responsiveness, which reflects intravascular volume status. Aortic V_peak_ serves as a non-invasive indicator of stroke volume variability.^10, 11^ PLR-induced >10% CI variability reflects fluid responsiveness and intravascular volume deficit.^11^ Changes in V_peak_ following PLR correlate with fluid responsiveness and preload dependency, making it an important parameter for assessing circulatory volume status.^12^ PLR avoids unnecessary fluid administration by identifying true volume-responsive patients. It is particularly useful in paediatric patients, where baseline hemodynamic values may fluctuate, and traditional static markers are less predictive.^13^

This study tested the hypothesis that preoperative fasting time has an impact on intravascular volume. The primary outcome was the relation between preoperative fasting time and CI variability after anaesthesia, induction in paediatric patients.

## Methods

### Study Design

This prospective, observational, single-center trial was conducted after receiving approval from the Institutional Ethics Committee of Marmara University Faculty of Medicine Clinical Research (approval no.: 09.2017.669, date: 08.12.2017) in accordance with the principles outlined in the Declaration of Helsinki.

**Figure 1:**
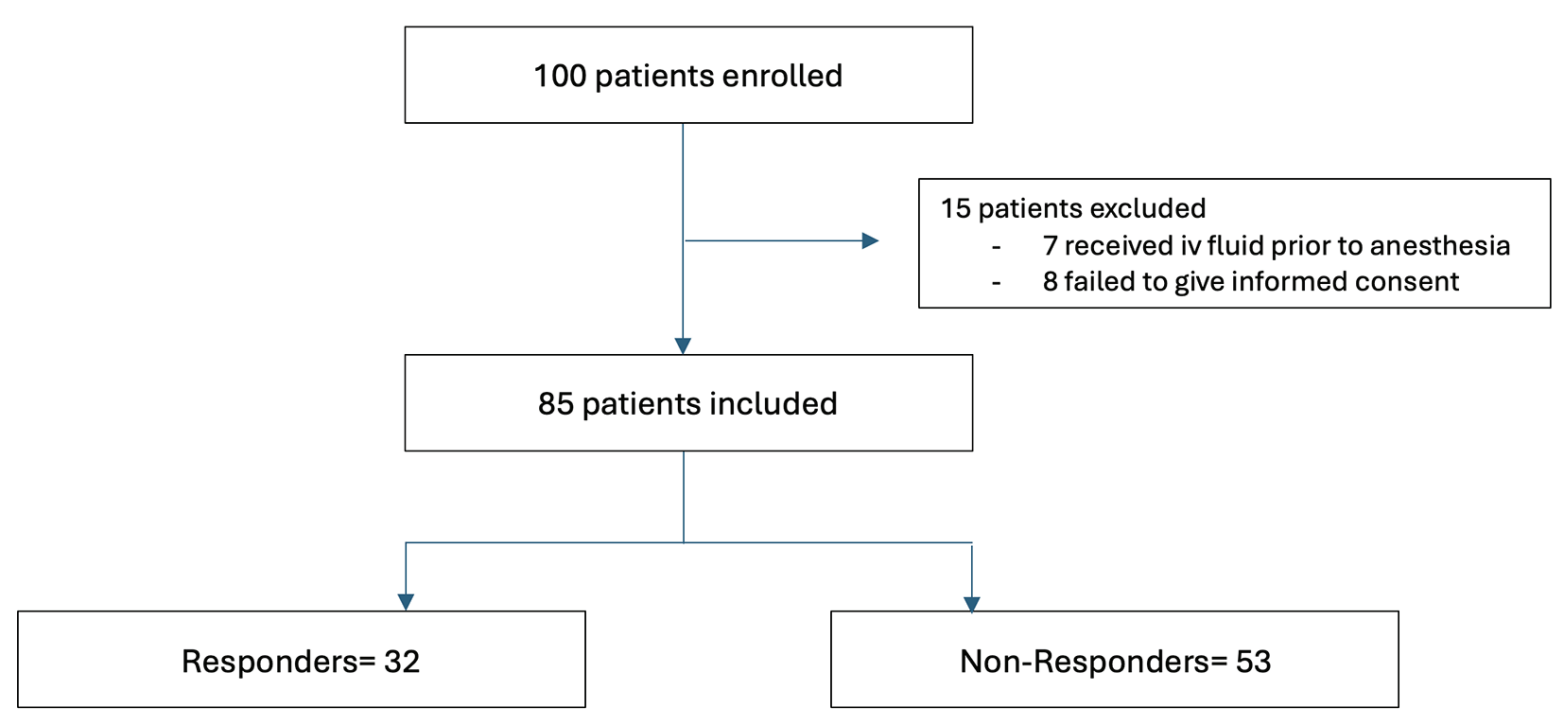
Flow chart

The trial was registered prior to patient enrollment at clinicaltrials.gov (https://clinicaltrials.gov/). Written informed consent was obtained from all patients’ legal representatives included in the study. The flow chart of the study is shown in Figure 1.

Patients over 5 years of age and with American Society of Anesthesiologists (ASA) physical status I, scheduled as outpatients for surgery, were asked to enroll in the study. Paediatric patients, who were otherwise healthy except for the need for surgery and who did not receive intravenous (IV) fluid prior to anaesthesia induction, were included. Patients who failed to meet inclusion criteria and refused to give informed consent were excluded.

### Anaesthesia Management

In the operating room, patients were monitored with an electrocardiogram, non-invasive blood pressure monitoring a pulse oximeter, monitors for anaesthetic gases, and a carbon dioxide (CO_2_) analyzer. Anaesthesia, was initiated through an IV route, with 1 µg kg^-1^ of remifentanil hydrochloride, 5 mg kg^-1^ of sodium thiopental, and 0.6 mg kg^-1^ of rocuronium bromide. Following induction, the patient’s airway was secured via tracheal intubation, and a maintenance dose of 2% sevoflurane in a mixture of 50% nitrous oxide and 50% oxygen was administered. Mechanical ventilation was controlled by the auto-flow mode of the Draeger Primus ventilator, with tidal volumes set at 8 mL per kg^-1^ of body weight. The respiratory rate was adjusted to maintain end-tidal CO_2_ between 35 and 45 mmHg, and a PEEP of 4 cm H_2_O was used for respiratory support. To keep the patient warm throughout the surgery, the active air warming system was employed.

### Protocol

After tracheal intubation, hemodynamic stability had been maintained with the aim of keeping a heart rate (HR) change of less than 10% for three minutes. Then, measurements were performed. The baseline measurements (T1) were recorded with the patient in the semi-recumbent position. Next, the PLR maneuver was carried out by placing the patient in the supine position and simultaneously raising the patient’s leg to 45°; after 1 minute (T2), a second set of measurements was recorded. In every stage, CI, CO, and aortic V_peak_ were measured; HR, CI, and mean arterial pressure were recorded. Cardiac measurement was performed through the suprasternal notch, via the Ultrasonic CO Monitor (USCOM) system by the same operator. Each parameter was measured consecutively three times, and the mean value of these three measurements was recorded.

An increase in CI (∆CI) >10% following PLR is considered indicative of fluid responsiveness and suggests a functional intravascular volume deficit. Patients were termed fluid responders (Rs) if ∆CI of >10% was obtained after PLR, or non-responders (NRs) if ∆CI was <10%.

### Statistical Analysis

Data were evaluated for normality of distribution and reported as mean, standard deviation (SD), median, 25^th^ and 75^th^ interquartile, frequency, percentage, and minimum and maximum range. The independent t-test was employed to compare the means between two unrelated groups, and paired samples t-test was used to compare the means of two related groups. Qualitative data was compared using the Pearson chi-square test. Receiver operating characteristic (ROC) curves were built, sensitivity and specificity of variables were calculated for various values, and the value with the highest Youden index was taken as a cut-off point. Spearman correlation coefficient was used to test the association between variables. Statistical significance was defined as *P *< 0.05.

Sample size was calculated based on a previous adult study investigating the functional intravascular volume deficit in patients before surgery.^14^ Flow time corrected (FTc) data of this study were taken into consideration. The FTc rise from 288 (±45) ms to 316 (±39) ms (before and after stroke volume maximization) was suspected to be significant. Accordingly, the minimum sample size required was 82 at an alpha level, and a beta level of 0.05 and 0.20, respectively. Based on possible dropouts, a total of 100 patients were planned for inclusion.

## Results

As shown in Figure 1, a total of 100 paediatric patients with ASA I, scheduled for outpatient surgery, were initially assessed for eligibility. Fifteen patients were subsequently excluded due to either the preoperative administration of IV fluids or their declining participation. A total of 85 patients were included. There were 32 R and 53 NR. The patient characteristics of two groups were comparable in terms of age, gender, height, and body weight. The mean age of the participants was 7.82±2.43 years. The mean fasting time was 10.95±2.84 hours. The mean fasting time for Rs was 11.53 hours, with a (SD) of 2.61, while NR had a mean fasting time of 10.6 hours, with a SD of 2.93. This difference was not statistically significant, with a* P* value of 0.145 (Table 1).

The hemodynamic parameters before (T1) and after (T2) PLR are shown in Table 2. HR significantly decreased after PLR in both R and NR (*P *< 0.001, *P *< 0.001, respectively), but showed an insignificant difference between the study groups. Mean arterial pressure values were comparable among the groups at all study time points. CO was significantly lower in R than in NR in T1 (*P*=0.043). CO significantly increased after PLR in R (*P *< 0.001) but remained similar in NR. ∆CO was significantly higher in R (0.89±0.56) than in NR (-0.12±0.49) (*P *< 0.001). V_peak_ was comparable among the groups in T1. Rs had a significantly increased V_peak_ in T2 (*P *< 0.001), and ∆V_peak _was significantly higher in Rs (0.24±0.17) compared to NRs (0.03±0.13) (*P *< 0.001). The optimal cut-off value for ∆V_peak _is >0.17, which corresponds to the highest Youden index of 0.63. It has a sensitivity of 84.9% and specificity of 78.1%. The area under ROC (AuROC) curve is 0.872 [95% CI (0.784, 0.961), *P *< 0.001]. Upon evaluating the relationships between fasting time and various cardiac measurements, including CI, CO, and V_peak_, fasting time showed no significant correlation with parameters (Table 3).

## Discussion

The objective of this study was to evaluate the impact of preoperative fasting duration on intravascular volume status in paediatric patients undergoing elective surgery. Using dynamic hemodynamic assessment via PLR, we found that prolonged fasting had no significant effect on intravascular volume status, as determined by CI variability (∆CI), CO, and aortic V_peak_.

The actual duration of preoperative fasting in paediatric patients is often longer than current recommendations, varying between 11-13 hours.^15, 16, 17^ Guidelines suggest reducing the clear fluid fasting period to approximately 2 hours before surgery to minimize discomfort and lower the incidence of hypotension in the perioperative period. However in clinical practices, paediatric fasting guidelines are often not applicable, resulting in longer fasting periods than recommended, with a high incidence of discomfort due to hunger and thirst.^16^ The present study, which found an average fasting time of 11 hours fasting time revealed similar prolonged fluid abstinence times in children scheduled for outpatient surgery.

In this study, the patients having more than 10% increase in CI following PLR had comparable fasting times with NRs. There was no correlation between the duration of fasting and CO and V_peak_. A study has suggested that prolonged fasting (more than 6 hours for liquid and 12 hours for solid) may increase the risk of hypotension during anaesthesia induction, particularly in paediatric patients.^9^ A retrospective analysis was conducted focusing on clear fluids fasting times in children after implementing a 1 hour clear fasting rule. This study noted that longer fasting times, particularly those significantly exceeding the recommendations of guidelines, were associated with adverse events related to hypovolemia dehydration, such as difficulty in vein cannulations, electrocardiogram alterations, and episodes of hypotension.^16^ Our findings contrast with prior studies that have reported associations between fasting duration and intraoperative hypotension. However, these studies primarily evaluated blood pressure changes as a surrogate for intravascular volume depletion, without incorporating dynamic assessments of fluid responsiveness.

Dynamic parameters, rather than static ones, have proven to be more reliable for predicting volume responsiveness in mechanically ventilated children.^10^ In contrast to static indicators of preload, dynamic tests analyze the effect of a defined change in preload on stroke volume or CO, thus giving an indication of the patient’s position on the Frank-Starling curve. Dynamic indices, therefore, are better predictors of changes in preload on the steep portion of the Frank-Starling curve and have been validated in predicting the response to volume expansion in both adults and children.^18, 19^ CI changes following PLR can identify patients who will benefit from fluid administration.^20^ The effectiveness of the PLR test partly depends on the ability to significantly alter venous return by elevating the legs. In smaller children, particularly in infants and toddlers, the relative volume shift achieved by lifting the legs may not significantly change the preload due to their smaller blood volume and different body proportions. PLR induced CI changes reliably reflects fluid responsiveness in children older than 5 years of age,^20^ but under this group of age it cannot identify all Rs with certainty.^13^ This is the reason why study included children older than 5 years of age. Also, aortic ∆V_peak_ predicted fluid responsiveness with an AuROC of 0.872 in the present study, supporting the evidence that aortic blood flow velocity is a reliable index to predict fluid responsiveness in children.^11^ USCOM measuring CO via a probe applied to the suprasternal notch revealed stroke volume variation as a reliable index in the prediction of fluid responsiveness.^21^ The change in V_peak_ measured by USCOM showed a significant correlation with the measurements obtained through the apical 5-chamber view.^22^ In light of these findings, we measured dynamic indices using USCOM.

Given the similar fasting times of Rs and NRs, it was suggested that fluid responsiveness cannot be directly related to the abstinence of oral intake. One-third of the children in our study were resistant; the reason for this could be attributed to the vasodilatory effects of the anaesthetic agents during the induction period. CO is directly related to the so-called stressed volume of the circulation.^23^ For optimal cardiac preload, it’s essential to have an adequate portion of the blood volume under stress, which depends on the state of the intravascular volume (normovolemia) and the tension within the blood vessels (vasotension).^24^ One potential explanation for the lack of correlation between fasting duration and hemodynamic changes is anaesthesia -induced vasodilation, which significantly alters intravascular dynamics independent of fasting status. General anaesthesia induction and neuraxial anaesthesia lead to vasodilation, reducing the stressed volume and causing a functional intravascular deficit.^14, 25^ The presence of a functional intravascular deficit could lead to changes in the CI when a PLR test is performed. Muller et al.^27^ showed that blood volume assessed by a dynamic test did not decrease after preoperative fasting. As measurements were performed before anaesthesia, hemodynamics were not affected by anaesthesia induction. At this point, our study assesses the impacts on both the duration of fasting and anaesthesia by evaluating dynamic parameters following anaesthesia induction. Thus, in our study population, any fasting-related reduction in circulating volume may have been overshadowed by the hemodynamic effects of anaesthesia, explaining the absence of a significant correlation between fasting time and fluid responsiveness.

Children exhibit unique physiological adaptations that allow them to maintain hemodynamic stability despite fasting-related changes in intravascular volume. Unlike adults, where stroke volume plays a dominant role in CO regulation, young children primarily rely on HR adjustments to preserve CO. In paediatric patients, stroke volume is relatively fixed due to less compliant ventricles, meaning HR plays the primary role in adjusting CO^23^. Even if fasting led to mild intravascular depletion, children may compensate by increasing HR, thereby preserving CO and preventing a detectable change in CI or CO.^19^

### Study Limitations

While our study provides important insights into fasting and hemodynamics in paediatric patients, certain limitations should be acknowledged. Primarily, it was conducted in a single institution, which may affect the generalizability of the findings. Additionally, the study included only children who were older than 5 years and were otherwise healthy apart from their surgical requirements. This selection criterion may restrict the applicability of our results to younger children, such as infants and toddlers, as well as to children with other comorbidities. Our study assessed volume status after anaesthesia induction, which means we could not evaluate fasting-induced changes before anaesthesia. While we assessed intravascular volume status, we did not measure interstitial fluid shifts, which could also play a role in perioperative hemodynamics.

Future studies may focus on a wider age range of children and those with cardiac, renal, and pulmonary comorbidities to assess the impact of fasting time on intravascular volume status more comprehensively and monitor longitudinal hemodynamic parameters before and after anaesthesia induction.

## Conclusion

In conclusion, preoperative fasting time had no effect on intraoperative intravascular volume. Anaesthesia-induced vasodilation and paediatric compensatory mechanisms (HR-driven CO maintenance, sympathetic activation) likely mitigate any fasting-related hemodynamic effects, explaining the lack of correlation between fasting time and fluid responsiveness. These findings emphasize the importance of using dynamic assessments over static indicators when evaluating perioperative fluid status and highlight the need for further research on fasting and hemodynamic adaptations in paediatric patients.

## Ethics

**Ethics Committee Approval:** The ethical approval was obtained from the Institutional Ethics Committee of Marmara University Faculty of Medicine Clinical Research (approval no.: 09.2017.669, date: 08.12.2017).

**Informed Consent:** Written informed consent was obtained from all patients’ legal representatives included in the study.

## Figures and Tables

**Table 1. Patient Characteristics:** 

-	**Total (n = 85)**	**Responders (n = 32)**	**Non-responders (n = 53)**	***P* value**
	**Mean ± SD**	**Mean ± SD**	**Mean ± SD**	
Age	7.82±2.43	7.69±2.61	7.91±2.33	^a^ **0.690**
**Gender n (%)**	-	-	-	^b^ **0.637**
Female	27 (100)	9 (33.3)	18 (66.7)	-
Male	58 (100)	23 (39.7)	35 (60.3)	-
Height	124.36±18.11	124.59±20.54	124.23±16.68	^a^ **0.928**
Weight	27.33±9.72	29.59±11.84	25.96±7.99	^a^ **0.131**
Fasting time	10.95±2.84	11.53±2.61	10.6±2.93	^a^ **0.145**

a, Independent t-test; b, pearson chi-square test. SD, standard deviation

**Table 2. Hemodynamic Parameters Recorded Before (T1) and After (T2) PLR:** 

**Mean ± SD**		**Responders (n = 32)**	**Non-responders (n = 53)**	**^a^ *P*** **(inter group)**
		**Mean ± SD**	-	
HR	T1	110.63±20.05	110.04±14.66	**0.877**
-	T2	98.38±17.03	96.15±14.27	**0.519**
-	∆	-12.25±8.93	-13.89±7.83	**0.378**
-	**^c^*P* (inter group)**	< 0.001**	< 0.001**	-
MAP	T1	71.38±12.6	69.26±8.27	**0.403**
-	T2	69.16±13.21	68.98±7.18	**0.945**
-	∆	-2.22±9.48	-0.28±6.63	**0.272**
-	**^c^** *P*	0.195	0.757	-
CI	T1	2.91±0.81	3.58±0.94	0.001**
-	T2	3.8±1.08	3.44±0.82	**0.092**
-	∆	0.89±0.53	-0.13±0.44	< 0.001**
-	**^c^ *P***	< 0.001**	0.032*	-
CO	T1	2.93±0.81	3.39±1.12	0.043*
-	T2	3.81±1.16	3.27±0.97	0.023*
-	∆	0.89±0.56	-0.12±0.49	< 0.001**
-	**^c^ *P***	< 0.001**	**0.076**	-
V_peak_	T1	0.93±0.25	1.02±0.24	**0.101**
-	T2	1.17±0.27	1.05±0.21	0.029*
-	∆	0.24±0.17	0.03±0.13	< 0.001**
-	**^c^** *P*	< 0.001**	**0.055**	-

a, Independent t-test; c, Paired Samples t-test, *P < 0.05, **P < 0.01

PLR, passive leg raising; HR, heart rate; MAP, mean arterial pressure; CI, cardiac index; CO, cardiac output; V_peak_, aortic peak velocity ∆: change; SD, standard deviation

**Table 3. Correlation Between Fasting Time and Cardiac Measurements:** 

-	**∆CI**	**∆CO**	**∆V_peak_**
Fasting time	-	-	-
r	0.117	0.126	0.199
**^a^** *P*	**0.285**	**0.249**	**0.067**

a, Independent t-test; r, pearson correlation coefficient; ∆CI, change in cardiac index; ∆CO, change in cardiac output; ∆V_peak_, change in aortic peak velocity
